# Exosome-based immunotherapy as an innovative therapeutic approach in melanoma

**DOI:** 10.1186/s12964-024-01906-1

**Published:** 2024-10-31

**Authors:** Shabnam Babaei, Manouchehr Fadaee, Hajar Abbasi-kenarsari, Dariush Shanehbandi, Tohid Kazemi

**Affiliations:** 1 Immunology Research Center , Tabriz University of Medical Sciences, Tabriz, Iran; 2 Department of Immunology, Faculty of Medicine, Tabriz University of Medical Sciences, Tabriz, Iran P.O. Box: 5165683146; 3 Student Research Committee, Tabriz University of Medical Sciences, Tabriz, Iran; 4 Department of Immunology, Faculty of Medicine, Shahid Beheshti University of Medical Sciences, Tehran, Iran

**Keywords:** Melanoma, Exosome therapy, Drug delivery, Cancer vaccine, Drug resistance

## Abstract

The malignant form of melanoma is one of the deadliest human cancers that accounts for almost all of the skin tumor-related fatalities in its later stages. Achieving an exhaustive understanding of reliable cancer-specific markers and molecular pathways can provide numerous practical techniques and direct the way toward the development of rational curative medicines to increase the lifespan of patients. Immunotherapy has significantly enhanced the treatment of metastatic and late-stage melanoma, resulting in an incredible increase in positive responses to therapy. Despite the increasing occurrence of melanoma, the median survival rate for patients with advanced, inoperable terminal disease has increased from around six months to almost six years. The current knowledge of the tumor microenvironment (TME) and its interaction with the immune system has resulted in the swift growth of innovative immunotherapy treatments. Exosomes are small extracellular vesicles (EVs), ranging from 30 to 150 nm in size, that the majority of cells released them. Exosomes possess natural advantages such as high compatibility with living organisms and low potential for causing immune reactions, making them practical for delivering therapeutic agents like chemotherapy drugs, nucleic acids, and proteins. This review highlights recent advancements in using exosomes as an approach to providing medications for the treatment of melanoma.

## Introduction

Melanoma, the most deadly and invasive form of skin cancer, is steadily rising globally [1, 2]. Melanoma accounts for 1.7% of the world’s cancers. In 2020, about 325,000 individuals get melanoma worldwide, and 57,000 people died from this disease [3, 4]. However, the survival percentage of people with melanoma is still low despite the significant advancements in therapy strategies [5, 6]. The five-year rate of survival for local melanoma is 98.3%; for metastatic melanoma (MM), it is 16% [7]. Initial therapeutic strategies for MM include surgical excision, chemotherapy, and radiation. Surgery is generally designated for early-stage melanoma and can efficiently remove tumor tissues; nevertheless, it is an invasive procedure. Despite their effectiveness in eliminating cancer cells, chemotherapy and radiotherapy are not selective and can inadvertently damage healthy cells, leading to severe consequences [8]. Before the beginning of immunotherapy for the management of MM, results were typically unpleasant despite the utilization of numerous cytotoxic drugs and compounds. The median survival for patients with incurable metastatic disease was between 6 and 9 months [9, 10]. In fact, immunotherapy, a well-established therapeutic approach, reduces harm to healthy cells and is regarded as the primary treatment for MM [8]. In recent decades, there has been significant progress in understanding the causes of melanoma and developing targeted therapies for treating it, such as targeted therapy and immune checkpoint inhibitors (ICIs). This has led to a significant shift in the way melanoma is treated [11]. However, despite these recent advancements, numerous patients now receiving immunotherapy experience cancer progression, emphasizing the continuing demand for the treatment of late-stage melanoma. Therefore, there is a need to investigate new methods for the treatment of melanoma. Utilizing exosomes to treat cancer is one of the most recent immunotherapy approaches. Exosomes can be used as potential cancer indicators, low-toxicity immunomodulators, or a safer and more effective way to administer anti-cancer medications [12]. Exosomes are increasingly utilized in biomedicine worldwide and have become essential contributors to developing more efficient treatment approaches for various diseases. Several fields can use them, such as immunological research, gene therapy, vaccine delivery systems, tissue regeneration, and as biomarkers in the detection and treatment of diverse diseases, including cancer and neurological disorders [13]. This review presents the recent advances in melanoma therapy, with a particular focus on exosome-based immunotherapy.

## Current therapy methods in melanoma

**Fig. 1:**
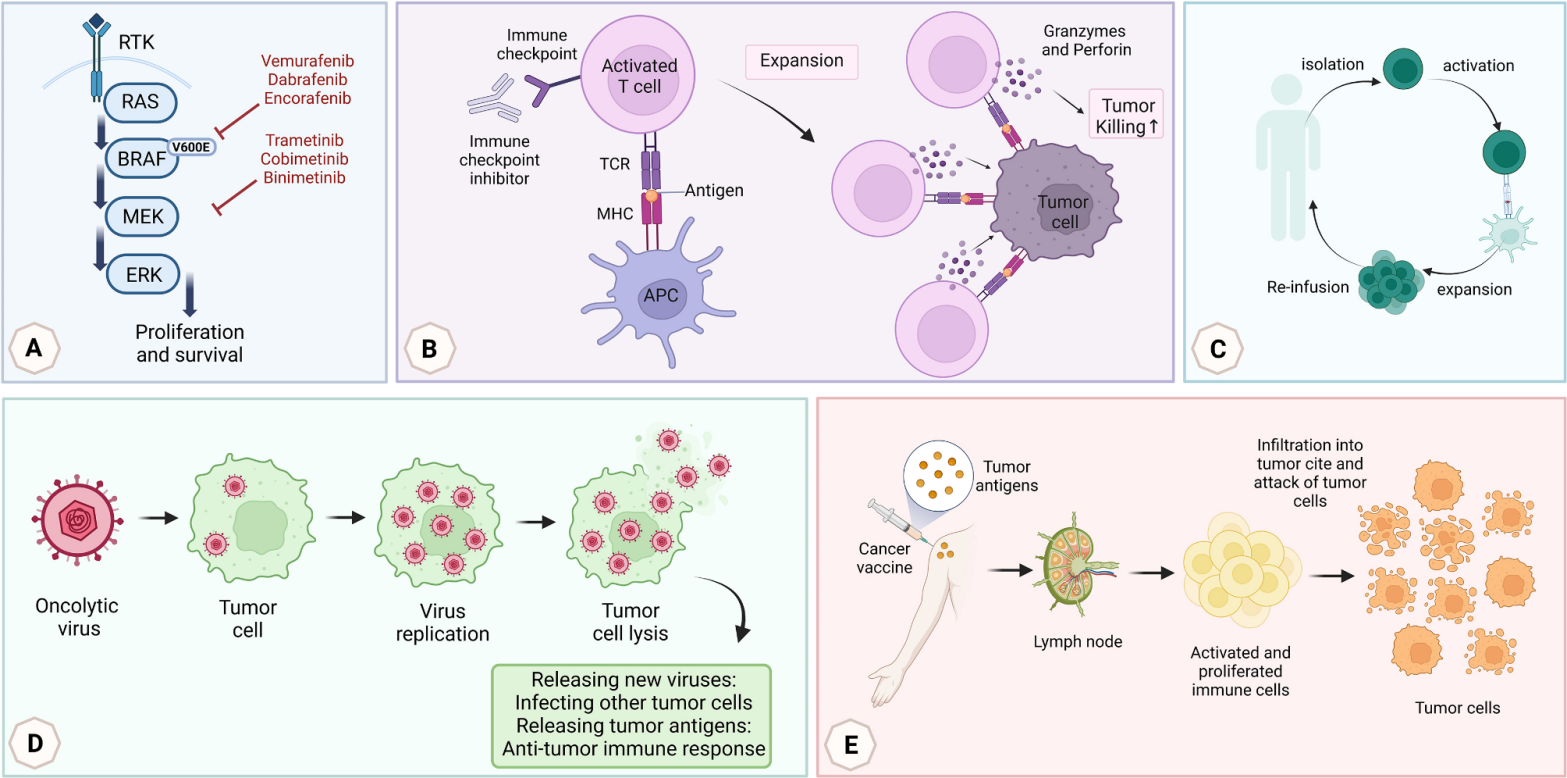
Current therapy methods in melanoma. (**A**) Targeted therapy, (**B**) Immune checkpoint inhibitors, (**C**) Adoptive cell therapy, (**D**) Oncolytic virus therapy, (**E**) Cancer vaccine (created by bio render)

This section briefly discusses melanoma cancer treatment options that are more effective and have fewer side effects than techniques like chemotherapy, surgery, and radiotherapy (Fig. 1).

### Immune Checkpoint Inhibitors (ICIs)

PD-1 and CTLA-4 are molecules involved in immunological checkpoint regulation. They play a part in suppressing paths that activate T cells, which are crucial for maintaining immune tolerance. PD-1 exists on the surface of activated T cells, B cells, Natural killer (NK) cells, and Monocytes. By binding to its ligand Programmed cell death-ligand 1 (PDL-1), PD-1 prevents the transmission of signals from the T cell receptor, thereby limiting the activation of T cells and the secretion of inflammatory cytokines. CTLA-4 is a protein homologous to CD28, expressing on regulatory T cells, where it suppresses signals to T cells [14].

#### Anti-CTLA-4 therapy

Immune responses against tumors have extensive links to CTLA-4, which plays a significant role in immunological tolerance. The interaction between B7 on antigen-presenting cells (APCs) and CTLA-4 triggers a series of inhibitory signaling events, leading to the inhibition of T-cell activity [15]. Antibodies targeting CTLA-4 can impede the interaction between CTLA-4 and B7, hence reinstating the functionality of T cells inside the antigen-presenting fraction (Fig. 1, B) [16]. Lang et al. conducted a trial involving 40 patients who received vemurafenib at a dose of 960 mg twice daily (BID) and another 40 patients who were treated with intravenous ipilimumab at a dose of 3 mg/kg body weight every three weeks for a total of four infusions. The average length of treatment with vemurafenib was 11.4 months. 32.5% of patients needed a decrease in the dosage, and 12.5% had to stop treatment early because of side effects. Among the patients treated with ipilimumab, 47.5% completed all four infusions. However. 32.5% received fewer infusions due to side effects, and 20% had their infusions reduced because of significant tumor growth. The ipilimumab group had a survival rate of 42.5% after 12 months and 35.5% after 24 months. In comparison, the vemurafenib group had a survival rate of 38% after 12 months and 25.5% after 24 months. 32.5% of patients (57.7% treated with ipilimumab and 42.3% treated with vemurafenib) achieved long-term survival, defined as surviving for at least 24 months [17]. In 2019, a study examined the treatment of patients with metastatic or incurable melanoma using either ipilimumab or dacarbazine. The control group consisted of 175 patients who received dacarbazine and 289 patients who received ipilimumab. Within the control group, a total of 41 patients were administered dacarbazine in combination with a platinum drug and other cytotoxic medicines. The one-year OS rate for dacarbazine was 18.9%, the two-year OS rate was 7.4%, and the three-year OS rate was 4%. In contrast, the corresponding OS rates for ipilimumab were 46.5%, 29.8%, and 24.4%, respectively [18].

#### Anti-PD-1/PD-L1 therapy

PD-1 is another regulatory molecule on the surface of many immune cells. The interaction between PD-1 and its ligands PD-L1/L2 on cancer cells or APCs inhibits the T-cell receptor signaling pathway, suppressing the immune system’s reaction [19]. The overexpression of PD-L1 has been observed in various malignancies, including melanoma, leading to the ability of cancer cells to evade the immune system. Blocking PD-1 restores the immune system’s ability to recognize and destroy cancerous cells (Fig. 1, B) [20]. The KEYNOTE-006 study compared the anti-PD-1 monoclonal antibody pembrolizumab with ipilimumab for treating advanced melanoma. The results showed that pembrolizumab was more likely to achieve PFS (hazard ratio 0.58) and OS (hazard ratio 0.63). In addition, Pembrolizumab had a lower incidence of toxicity, with a grade 3 adverse reaction rate of 13.3% compared to 19.9%. Pembrolizumab received FDA approval for the therapy of inoperable or aggressive melanoma in the primary situation [21]. A double-blinded phase III study was also conducted to examine melanoma patients who had not previously received any treatment. Nivolumab, another anti-PD-1 monoclonal antibody, had a five-year OS measure of 39%, PFS of 28%, and an ORR of 42%. In contrast, dacarbazine had these rates of 17%, 3%, and 14%, respectively. These findings validate the substantial advantage of nivolumab over dacarbazine for all outcomes and contribute to the growing body of evidence supporting the long-term survival of patients treated with nivolumab alone [22].

Anti-PD-1 medications can lead to immune-related adverse events (irAEs) and other adverse reactions. However, the occurrence of severe events (grades 3–5) has decreased compared to ipilimumab, with rates varying from 8 to 16% in patients given either nivolumab or pembrolizumab [23]. Moreover, several trials have examined the use of combination therapy of ICIs for melanoma. For instance, the combination therapy of nivolumab and ipilimumab has been accepted as the initial regimen for dual checkpoint blocking in patients with both BRAF-wild-form melanoma and BRAF-mutated ones due to the enduring and consistent survival benefits along with a satisfactory safety profile [24].

#### Anti-lymphocyte Activation Gene 3 (LAG-3) therapy

LAG-3 is found on T cells and serves as the third receptor for immunological checkpoint co-inhibition, which holds potential for use in immunotherapy for cancer. Due to its greater affinity for major histocompatibility complex (MHC) II compared to CD4, it suppresses the activation of T cells [25]. Although LAG-3 inhibition alone has limited effectiveness in treating malignancies, the combination of anti-LAG-3 and anti-PD-1 has shown significantly improved therapeutic action in various mouse cancer models, including melanoma [26]. Relatlimab, the most potent anti-LAG-3 antibody, has undergone evaluation in conjunction with nivolumab during a phase II-III trial, including individuals with uncontrolled metastatic or inoperable melanoma. The trial results demonstrate that the PFS after receiving a combination of relatlimab and nivolumab therapy is superior to the PFS achieved with nivolumab alone (the median PFS was 10.1 and 4.6 months, respectively) and close to the PFS achieved with ipilimumab and nivolumab in the past. But, grade 3/4 treatment-related adverse events appeared in 18.9% of those participating in the combined arm and 9.7% of participants in the nivolumab arm [27].

### Targeted therapy

Targeted therapy, which blocks particular molecules or routes of signaling, is a crucial treatment strategy for metastatic skin melanoma. The purpose of targeted therapy is to inhibit the mutant genes to prevent their far-reaching proliferation. Numerous mutations, such as BRAF and neuroblastoma ras viral oncogene homolog (NRAS) mutations, have an oncogenic impact through the activation of the Mitogen-activated protein kinases (MAPK) pathway, which in turn leads to an enhanced proliferation of tumor cells. Melanoma patients with these two gene mutations are often treated with targeted therapy (Fig. 1, A) [28, 29].

#### Mono-inhibitors of BRAF and MEK

The Val600 codon in the kinase domain of BRAF is responsible for over 97% of the observed mutations. Among these, 90% involve the substitution of the amino acid valine with glutamic acid (BRAFV600E) [30]. Several BRAF inhibitors have been approved, such as vemurafenib, dabrafenib, and encorafenib [28]. Additionally, inhibitors targeting downstream components of the signaling pathway, like MEK, have been created to effectively prevent a broader range of upstream cancer-causing mutations [31]. MEK inhibitors include trametinib, cobimetinib, and binimetinib [28]. This type of treatment has various side effects, including skin poisoning, photosensitivity, arthralgia, and cardiovascular poisoning. However, its most significant limitation is that, although most patients initially respond well, the tumor’s evolving resistance pathways eventually cause the response to diminish [28, 29]. Monotherapies with BRAF/MEK inhibitors also raise the chance of abnormal MAPK pathway signaling in nonmelanoma cells, which increases the incidence of RAS-mutant cancer reactivation and the emergence of additional cancer [32].

#### Combination inhibitors of BRAF and MEK

Combination therapy using BRAF and MEK inhibitors increases the effectiveness of these drugs and postpones the emergence of resistance. This type of combined therapy decreased the probability of additional cancers caused by the treatment without raising the incidence of other severe toxicities [32]. Currently, the FDA and European Medicines Agency (EMA) have approved three different BRAF/MEK combination treatments, which include dabrafenib plus trametinib, cobimetinib plus vemurafenib, and binimetinib plus encorafenib [29]. In a double-blind phase III clinical trial, unresectable melanoma patients with the BRAF Val600 mutation were randomly assigned to receive either an injection of dabrafenib (150 mg orally twice daily) plus trametinib (2 mg orally once daily) together, or dabrafenib and a placebo. The group treated with the combination drug had a better OS than the single drug (median OS: 25.1 versus 18.7 months, respectively). In both groups, the treatment-related adverse event and Grade 3/4 adverse event were almost identical [33]. Similarly, the combination of kencorafenib and binimetinib had a better tolerability profile than those treated with encorafenib or vemurafenib alone for advanced (stage IIIB/C or IV) skin melanoma with mutations in BRAF V600. PFS and OS have also improved in a combined group compared to each monotherapy group [34, 35]. In addition, in a Phase 1b study involving patients with BRAF mutant melanoma, 129 individuals were administered ten dosing regimens that combined vemurafenib and cobimetinib. Confirmed objective responses were observed in 15% of the 66 participants who had recently experienced progression on vemurafenib, with a median PFS of 2.8 months and were observed in 87% of the 63 participants who had not previously received a BRAF inhibitor, with 10% achieving a complete response; the median PFS was 13.7 months. Melanoma patients respond better to this combination of treatments; however, those who develop resistance still face a challenge [36, 37].

#### Combination of immunotherapy and targeted therapy

ICIs and targeted therapy may work in tandem as a therapeutic addition. Although most responses to BRAF and MEK inhibitors are temporary, they are associated with higher objective response rates [38]. However, in ICIs, response rates tend to be lower, and they provide a higher level of durability [39]. Atezolizumab, a PD-L1 inhibitor, along with the BRAF inhibitors vemurafenib and cobimetinib, had an efficiency for the treatment of melanoma patients with BRAF V600 mutations. Compared to vemurafenib plus cobimetinib, this combination therapy significantly extended the PFS to 4.5 months and decreased the relative risk of progressing or dying by 22% [40]. Overall, ICIs with targeted therapy can prolong response duration, but more research is needed.

### Cancer vaccines

Various approaches attempted to develop a potent vaccination for melanoma, including melanoma-cell-targeted vaccinations, anticancer lymphocytes, dendritic cell (DC) vaccinations, vector-based vaccines, peptide-based vaccines, and messenger ribonucleic acid (mRNA)/ Deoxyribonucleic acid (DNA) vaccines [32, 41]. Cancer vaccines’ primary objective is to stimulate immune system reactions by activating effector T cells against tumors. The vaccines diminish the volume of tumors and, thus, establish immunological memory (Fig. 1, E) [42]. Although some melanoma cell-based vaccines have shown promise in in vitro investigations, they lack therapeutic effectiveness to date [32].

DC vaccines elicit targeted immune responses capable of selectively eradicating specific cells [43]. Vaccines generated from monocyte-derived DCs pulsed with tumor extracts can influence the tumor microenvironment (TME) and facilitate the conversion of a “cold” cancer to a “hot” cancer. These are administered by directly injecting activated or altered DCs into the area of the tumor [44]. DCs stimulate the activation and recruitment of CD8 + T cells in MM patients [45]. Although these types of vaccines have exhibited immunogenicity in mouse models, their effectiveness has not yet been proven in human trials, most likely because of the immunosuppressive characteristics of the TME [46]. For these reasons, DC vaccination is considered a potential target for combination with PDL1/PDL1 immune checkpoint inhibition.

Vector-based vaccines employ recombinant viruses as vectors to directly transport antigens from tumor transgenes to APCs. This way, the transgenes activate T cells through MHC I and MHC II. These vaccines have been extensively utilized as therapeutic gene vectors, functioning as oncolytic substances due to their capacity to stimulate the body’s immunity against tumor cells by generating immunomodulatory mediators such as cytokines [41, 47].

Peptide vaccines provide fragments of tumor-specific (e.g., melanoma antigen recognized by T cells 1 (MART-1/MelanA), tyrosinase (tyr), and glycoprotein 100 (gp100) or tumor-related antigens (Melanoma-associated antigen 1 (MAGE-A1), NY-ESO-1, and MAGE-A10) that can be given by APCs to stimulate the activation of effector T cells. While experimental studies are promising, vaccinations using short peptides have shown low efficacy in clinical settings, even when combined with stimulating adjuvants [48].

DNA vaccines provide advantages as anticancer therapies, demonstrating immunogenicity and safety in clinical trials, although they lack substantial efficacy [41]. A phase I clinical trial tested a murine tyr DNA vaccine for individuals with melanoma. The results showed that the vaccine was safe and triggered a tyr-reactive reaction in 40% of the patients, with only grade 1 toxicity at the injection location [49].

mRNA vaccines against cancer offer the benefits of swift and cost-effective manufacturing. Unlike DNA vaccines, they do not exhibit significant adverse consequences, such as incorporation into the individual’s genome. These vaccines possess the capability to elicit humoral as well as cellular immune reactions [50]. However, the development of mRNA vaccines for cancer has made little progress due to challenges related to the instability of mRNA, insufficient effectiveness in cells, and high intrinsic immunogenicity [51].

### Adoptive Cell Therapy (ACT)

ACT employing tumor-infiltrating lymphocytes (TILs) is a cell-based immunotherapeutic approach that entails the isolation of lymphocytes from cancerous tissue, augmentation of this population, and subsequent reintroduction into the patient’s body, enabling them to identify and eliminate cancer cells (Fig. 1, C) [52]. Phase II research with Lifileucel, an autologous, centrally manufactured TIL product, demonstrated the efficacy and durability of responses in strongly pretreated individuals. The participants in this trial were advanced melanoma patients who had previously been treated with ICIs and targeted therapy. Patients received a single injection of lifileucel, a nonmyeloablative lymphodepletion protocol, and up to six dosages of high-dose IL-2. This finding suggests that Lifileucel has the chance to become an emerging standard treatment for melanoma patients with failed other treatments. All patients encountered at least one treatment-related adverse event; however, grade 3/4 adverse effects declined quickly, with no lifileucel-related major adverse events recorded after 6 months and no return of irAEs associated with past ICI therapy [53]. Also, Patients who received TIL treatment had a considerably longer median PFS (7.2 months) than those who received anti-PD-1 treatment (3.1 months). Nevertheless, the patients who had TIL therapy experienced a significantly greater incidence of grade ≥ 3 effects, with an incidence of 100% compared to 57% for patients who received ipilimumab [54].

The ACT combination with ICI therapy is also under investigation. The ongoing experiment is exploring the utilization of ACT in combination with anti-PD-1 treatment [55, 56]. Although ACT has not yet received FDA approval for the MM therapy, it shows promise as a therapeutic option for patients who have not responded to conventional ICI medications.

### Adjuvant therapy

A variety of systemic therapies tested melanoma treatment with adjuvants. Systematic adjuvant therapy aims to reduce the chance of recurrence, death, or both for high-risk melanomas following surgery [57, 58]. For instance, IFN-α injection increases cancer immunogenicity by encouraging the development of immunity against the tumor. IFN-α hinders the growth of melanoma cells and reduces their synthesis and secretion of vascular endothelial growth factor (VEGF), which in turn lessens the formation of capillary angiogenesis surrounding the tumor [59]. Further extensive research has demonstrated that high-dose IFN-α2b (HDI) therapy for melanoma enhances relapse-free survival (RFS) and, to a lesser degree, OS [60–63]. Subsequently, pegylated IFN was used to improve tolerance to IFN. The fewer weekly injections and longer half-lives of pegylated IFN result in slightly better side effects than HDI [64]. Although IFN is no longer the first-choice medication for most patients, it might still be helpful as a supplemental immunostimulatory drug to improve the therapeutic outcomes of other immunotherapies [32].

The other advancement of adjuvant therapy is ICIs in an adjuvant setting.

In a phase III trial that followed a randomized, double-blind design, a total of 906 patients who had undergone a complete excision of stage III/IV melanoma assigned randomly to get either ipilimumab (10 mg/kg) or nivolumab (3 mg/kg), with the primary objective of RFS. The 1-year RFS was significantly greater in the group getting nivolumab (70.5%) compared to the group getting ipilimumab (60.8%) (*P* < 0.001). Nivolumab had a lower incidence of treatment-related adverse events compared to ipilimumab in the safety evaluation. 14.4% of patients treated in the nivolumab arm and 45.9% in the ipilimumab arm experienced grade 3/4 adverse events, leading to termination rates of 3.5% and 30.0%, respectively [65]. Additionally, nivolumab had a 5-year OS of 76%, compared to 72% in ipilimumab [66]. Pembrolizumab also enhanced distant metastasis-free survival (DMFS) (65.3 vs. 45.4%) as well as recurrence-free survival (59.8 vs. 41.4%) when compared to the group receiving placebo during an average follow-up period of 42.3 months. These improvements were observed in a phase 3 trial which inducted on 1019 melanoma patients with stage IIB/IIC who underwent entire resection, which was approved in both the United States and Europe [67]. Another study revealed that the occurrence of more significant toxicity was more prevalent with IFNa2b and ipilimumab in comparison to pembrolizumab, with rates of 66% and 43%, respectively, as opposed to 17% [68].

Current research has not demonstrated any improvement in OS. Likely, factors such as post-relapse therapy, insufficient surveillance, or unresolved biochemical and immunological aspects may have an influence. More than 30% of patients with high-risk melanoma who received adjuvant therapies experienced a recurrence of the disease within two years after surgery [69].

### Oncolytic virus therapy

The primary location of melanoma lesions on the skin and their tendency to spread locally makes it possible to treat these lesions directly with lower procedural risks. Talimogene laherparepvec (T-VEC) is an example of an intra-lesional treatment. T-VEC is a genetically altered oncolytic Herpes simplex virus 1 (HSV-1). The development of T-VEC involved the removal of the ICP34.5 gene, which encodes the herpes neurological virulence factor. This deletion led to the loss of the virus’s capacity to reproduce in neurons while retaining its capacity to enter and increase in cancerous cells (Fig. 1, D). Furthermore, the virus has undergone modifications with the introduction of tumor-specific inducers, leading to the development of tumor-specificity [70].

The viral genome was further modified to incorporate the gene encoding human granulocyte-macrophage colony-stimulating factor (GM-CSF), which stimulates DCs and enhances the presentation of cancerous antigens to T lymphocytes. Pre-clinical experiments have demonstrated that HSV-1 strains carrying GM-CSF exhibit an increased immune response compared to those without GM-CSF production [15]. Furthermore, combining T-VEC with either ipilimumab or pembrolizumab is highly effective in treating MM while also maintaining a safe and well-tolerated therapeutic profile. In a phase II study, 98 MM patients were treated with T-VEC combined with ipilimumab, while 100 patients were treated with ipilimumab alone. Regarding those with stage IIIB/IIIC/IVM1a MM, the ORR was 44% in the combination group versus 19% in the ipilimumab group; further, the ORR for those with stage IVM1b/IVM1c disease was 33% and 16%, respectively. 98% in the combo group and 95% in the ipilimumab group had ≥ 1 treatment-related adverse event. The incidence of ≥ 3 adverse events was 45% and 35%, respectively [71]. The study on the systemic use of oncolytic virus therapy is now underway. Nevertheless, maintaining viral titers that can effectively induce an anticancer response following systemic treatment has presented difficulties in monotherapy with systemic oncolytic viruses [32].

## Exosome-based immunotherapy

Virtually all cell types release exosomes, nanovesicles ranging from 40 to 160 nm in size. Exosomes, which serve as mediators of intercellular interactions, transport lipids, DNA, proteins, and RNAs (including microRNAs (miRs)) from donor cells to target cells [72, 73]. Biological fluid samples (liquid biopsies) easily access the complex payload of exosomes because they are present in all bodily fluids [74]. They are originated from late endosomes and multi-vesicular bodies (MVBs) in cells and are released into the extracellular space upon MVB fusion with these cell membranes, they resemble parent tumor cells in both the morphology of their surfaces and the content of their lumens [75, 76]. Exosomes can release their cargo by either fusing with the membrane of the recipient cell or undergoing phagocytosis. The uptake of exosomes by target cells results in the transfer of their cargo, which subsequently influences the phenotype of those cells [77]. Using exosomes as a therapeutic tool for cancer therapy, though their application in human cancer therapy is still in need of more research, has demonstrated a great potential in laboratory and animal studies. Although immunotherapy has significantly advanced the field of cancer therapy and increased advanced patient survival rates, solid tumors do not respond well to these available immunotherapies. As opposed to solid tumors, hematologic malignancies exhibit a substantially greater response rate to immunotherapy due to different levels of specific immunoregulatory compounds, which significantly increases the interaction of immune cells and hematological tumor cells [78, 79]. The advancement of research about the efficiency of immunotherapy for solid tumors has been inefficient, mostly attributable to the following reason: (1) The impact of hypoxic and suppressive TME on T cell growth, differentiation, and effector function. (2) The impact of the extracellular matrix, tumor vasculature, and cancer-associated fibroblasts on the infiltration of therapeutic drugs and immune cells, T cell growth, and its cytotoxicity. (3) The impact of myeloid-derived suppressor cells (MDSCs), regulatory T cells (Tregs), and tumor-associated macrophages (TAMs) on suppression of immune cells in the TME [79, 80]. For instance, due to the tumor’s evasion of the immune system, Ipilimumab, a CTLA-4 inhibitor, has enabled 21% of patients to survive beyond 10 years, and PD-1 inhibitors assist 30% of patients with advanced cancer in achieving survival beyond 5 years [81, 82]. ICIs can also induce irAEs and other adverse effects [23]. Therefore, while available immunotherapies show considerable promise relative to prior cancer treatments, the challenges of drug resistance and limited efficacy indicate the necessity for the exploration of novel and more effective immunotherapeutic approaches for cancer treatment. Exosomes serve as the primary mediators of intercellular transfer of signals, playing a vital part in the intricate network of communication between tumor cells and immune cells. Exosomes, on the one hand, facilitate communication among immune cells, thus activating subsequent effector cells. On the other hand, they exhibit tumor-specific antigens to the immune cells, hence impeding the immunological evasion of tumor cells [83, 84]. Moreover, exosomes can easily penetrate the extracellular matrix of tumor tissue, remaining impervious to the TME, thus surmounting the obstacles associated with cell treatment [85].

The safety, effectiveness, and distribution of medicinal products are essential. So far, various technologies, including micelles, inorganic and polymeric nanoparticles, liposomes, etc., have been utilized for drug delivery [86]. Exosomes are appealing for delivering medications to address these issues due to their stability, biocompatibility, and low immunogenicity [87–89]. In particular, bioengineered exosomes effectively employ to selectively deliver anti-tumor solid medications (like RNAs and chemotherapy agents) to tumoral cells [90, 91]. As the exosome is a naturally occurring mediator, it possesses the innate capacity for permeability in cells, which enables it to pass through biological obstacles and even bypass the endosomal and lysosomal proteolytic routs, which improves delivery effectiveness and minimizes unintended effects [92]. Exosomes also create a protective environment for the enclosed cargo, shielding it from destruction and enhancing its durability [93]. The effectiveness of exosome-based immunotherapies in melanoma is summarized in Table 1.

In addition to using natural exosomes, we can engineer them to improve their therapeutic potential and boost the effectiveness of current agents. Over the past few years, researchers have devised diverse exosome modification processes, including genetic engineering, chemical alteration, and cargo packaging, to enhance their therapeutic efficacy [94]. Preconditioning donor cells with cytokines that boost immunity can also improve their exosomes’ production or therapeutic effectiveness [95]. For instance, exosomes produced from NK cells triggered by Interleukin (IL)-15 and IL-21 exhibited significantly enhanced cytotoxic activity [96]. Genetically modified techniques are effective for presenting specific ligands on the exosomal membrane. Parental cells can produce the target membrane-bound molecule via lentiviral packaging or transfection with a plasmid containing the gene of interest. Donor cells that overexpress certain proteins alter the membrane surface of the exosomes they produce [97, 98]. An outstanding example involves transfecting DCs with the plasmid vector pcDNA3.1(+) containing the PD-1 single-chain variable fragment gene, which facilitated the creation of membrane-localized anti-PD-1 antibodies. Exosomes extracted from these transformed cells subsequently regenerate exhausted CD8 + T cells, enhancing CTL reactions [99]. Exosomes can change chemically to reveal artificial or natural ligands on their surface, which demonstrated significant targeting efficiency for their receptors on cancerous cells [94]. These modifications facilitate the precise delivery of exosomes to target sites, such as cancer cells and immune cells, thereby minimizing adverse effects on surrounding cells while ensuring the specific transport of the intended payload to the target cell through receptor-ligand interaction.

### Exosome-based drug delivery

**Fig. 2:**
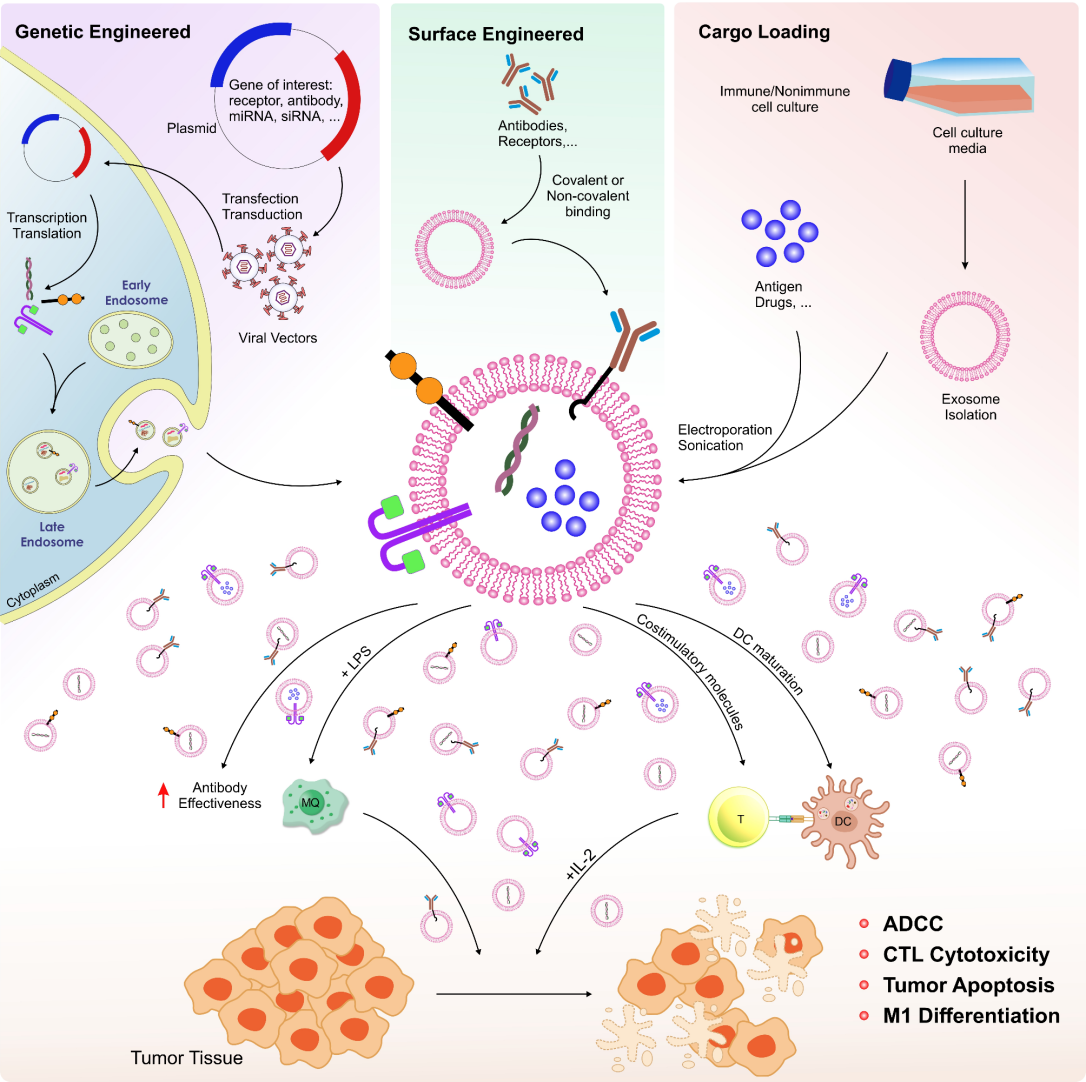
Exosome-based immunotherapy in melanoma. Exosome-based therapy in melanoma mainly employs three general methods. (1) Genetic engineering initiates the alterations, allowing the targeted gene to infiltrate the cell via a plasmid, multiply within it, and subsequently release it from the cell via exosomes. (2) Exosome surface modifications involve the use of linkers to introduce materials like antibodies and adhesive molecules onto the exosome’s surface, enabling the exosome to adhere specifically to the target cell. (3) The process involves importing cargo, such as chemotherapy drugs and antigens, and delivering them to the desired cell using mechanical and chemical methods. Modified exosomes can enter the tumor microenvironment and affect various immune cells. For example, in the presence of adjuvants, they can switch macrophages to M1, increase antibody efficacy and T-cell stimulation, enhance dendritic cell maturation, and ultimately increase the immune system’s response to a tumor.

Traditional cancer chemotherapy medications exhibit several limitations, including diminished bioavailability, restricted therapeutic efficacy, and ambiguous adverse effects. In the past few years, drug delivery methods have become an essential area of study. Drug delivery methods play a crucial role in augmenting the therapeutic effectiveness of anticancer medications while concurrently minimizing their systemic hazards through precise and targeted therapy [100]. Since exosomes are mainly involved in transferring chemicals between cells, it is reasonable to use them as a practical method for the transfer mechanism [101] (Fig. 2).

#### Drug-loading methods

There are various drug loading methods, each with its own set of advantages and disadvantages. They include incubation, physical loading techniques like electroporation, ultrasonic, and extrusion, as well as cell engineering approaches [102]. During incubation, the therapeutic agent penetrates the membrane of the exosome or cell based on a concentration differential. The benefits include easy operation, no need for specific equipment, maintenance of exosome integrity, and minimal damage to exosomes and medicines. This procedure involves loading a variety of medications [103, 104]. However, this method may lead to cell toxicity and influence the cell morphology [102, 105]. Electroporation is a technique in which, by applying an electric shock, the permeability of the cell membrane increases, and medications are rapidly delivered into the exosome [106]. Following drug loading, the exosome membrane promptly regains its structural integrity [107]. Electroporation is a simple method that has been widely used to encapsulate miR and small interfering RNA (siRNA). However, it can cause nucleic acid to precipitate or degrade and exosomes to clump together or damage, reducing the effectiveness of drug loading [105]. The ultrasound approach facilitates drug penetration into the exosome by mechanically breaching its membrane through shear forces [108]. While ultrasound may improve drug loading efficiency, it may compromise the exosomal membrane and lead to exosome aggregation [102]. Ultrasound and electroporation are effective comparing to other drug loading techniques, however they are best suited for small-scale lab application [109]. Extrusion is another method in which the membrane of exosomes is disrupted by an external force, enabling their combination with medications and the formation of new exosomes [110]. This approach has excellent drug loading effectiveness and produces uniformly sized exosomes. Investigators have not definitively established whether the mechanical force principle will alter the characteristics of the released exosome membrane [105]. Finally, these physical methods exhibit great drug-loading effectiveness, but the potential damage to exosome stability and integrity, specific technology needs, and constraints on the output scale limited them [102]. Cell engineering, as another method, involves altering donor cells using gene editing techniques or other methods to generate exosomes with particular targeted proteins on their cell membrane. This method is an extremely established and intricate methodology with low toxicity, which is used extensively for loading payloads into exosomes. The drawbacks of this approach include complex procedures and uncertainty surrounding the composition and quantity of exosomal components and cargo [102].

#### Application of exosome-based drug delivery in melanoma

Chemotherapy, despite its side effects, is a commonly used palliative treatment for advanced MM [111]. However, the majority of outcomes from chemotherapy medicines are temporary, as only about 1–2% of patients develop a long-lasting response to this therapy. Relevant research has shown that EV-based chemotherapy offers a survival advantage compared to chemotherapeutic drugs alone [112]. Patras et al. compared the effects of extracellular vesicles (EVs) derivatives from a murine melanoma model modified with polyethylene glycol (PEG) and loaded with doxorubicin (DOX) with liposomal DOX in B16.F10 mice. Both the EV and liposome groups intravenously administered a dosage of 2 mg/kg DOX to C57Bl/6 melanoma-bearing mice. Their analysis indicated that therapy with the mentioned dose of PEG-EV-DOX resulted in a more significant inhibition of tumor growth compared to liposome-DOX (76% vs. 51% inhibition in comparison to tumors in control, *P* = 0.0369). The two-fold enhancement in the therapeutic efficacy of PEG-EV-DOX may be attributed to its prolonged systemic circulation period, facilitated by the hydrophilic PEG covering and the smaller size of EVs, resulting in increased accumulation of DOX within the tumor and enhanced tumor-targeting capability. Nonetheless, the impact of prolonged administration of PEG-EV-DOX on melanoma tumors and the assessment of continuous exposure of mice to drug delivery systems remain unexamined and necessitate additional investigation [113]. Triptolide (TPL), another anticancer drug, has diverse biological and pharmacological properties that implicate in the treatment of cancer. Nevertheless, due to its insoluble nature and high systemic toxicity, the clinical use of this drug is limited [114]. A study investigated a TRAIL (TNF-related apoptosis-inducing ligand)-engineered delivery system that used exosomes from macrophages loaded with TPL. The highest level of TPL in the tumor was found in the TRAIL-exosome/TPL group (402.33 ± 6.80 ng/g) 4 h after the drug was given. This was 1.39 times higher than the exosome/TPL group and 7.94 times higher than the TPL alone group. In vivo, TRAIL-exosome/TPL sustained high drug residues in tumors for 24 h, showing that this modified delivery mechanism extended TPL circulation. Furthermore, TRAIL-exosome/TPL, in contrast to free TPL, did not induce any systemic toxicity, side effects, or bone marrow inhibition. So, this engineered drug delivery mechanism is safe and easily tolerated for treatment of tumors. Their results showed that TRAIL-exosome/TPL can increase cytotoxicity, impede tumor expansion and migration, and facilitate tumor apoptosis by activating both extrinsic and intrinsic apoptotic mechanisms [115]. Exosomes can transport genetic information or antibodies in addition to chemotherapeutic medications; Table 1 provides some examples of their use in melanoma cancer. For example, in ICIs, over 50% of cancer patients exhibit resistance to anti-PD1/PDL1 therapies due to the depletion of specific tumor-associated antigens, inadequate maturation of DCs, and insufficient activation and infiltration of CTLs [116, 117]. In a 2023 study, researchers developed a new gene-engineered exosome (PD1-Imi Exo) by integrating the PD1 gene and concurrently encapsulating the immune adjuvant imiquimod, which enhances the efficacy of ICIs therapy by reversing T lymphocyte exhaustion through DC activation and subsequently restoring T lymphocyte functionality. Moreover, PD1-Imi Exo is a naturally occurring protein that easily infiltrates the TME and exhibits a superior binding affinity for tumor cells compared to free PDL1 antibodies, hence enhancing the ICIs impact. Besides, the formulation is comparatively easy and requires future advancement for clinical use [118]. Researchers conducted another study on the melanoma cell line A375, which exhibits elevated ribonucleotide reductase regulatory subunit M2 (RRM2) expression [119]. The overexpression of RRM2 augments tumor angiogenesis and contributes to the metastatic and invasive potential of human cancer cells [120]. They engineered an arrow-tail RNA nanoparticle to provide folate RNA aptamer on the exosomal surface for M2 siRNA delivery in cancer therapy. Numerous human cancer cells express folate receptors highly. Consequently, folic acid modification on the exosomal surface can enhance the targeting efficacy of RNA nanomedicines and facilitate the delivery of therapeutic agents to specific cancer cells via exosomes [121]. Upon entrance into the cancer cells, siRNA inhibited RRM2 expression. This inhibition exhibited significant regression of melanoma development. The findings indicate that fully encapsulated exosomes with folate presentation and siRNA incorporation reduced tumor volume by 60%, compared to a 27% reduction with siRNA alone and a 48% reduction with folate alone [119].

### Exosome-based immunostimulation in melanoma

#### DC-Derived Exosomes (DEXs)

Tumor vaccines possess distinctive benefits in immunotherapy, such as the capacity to induce active immunity, establish a long-lasting immune memory, and harness the synergistic effects of multitarget therapy. Among these, DC-based vaccines stand out due to their potent antigen-presenting capacity, which makes them efficiently elicit specific immune responses to the target antigens. Nevertheless, there are numerous challenges with cellular vaccines, such as a limited amount of proliferation, intricate quality control, a confined source, and challenges with production and maintenance techniques [122, 123]. Exosome-based vaccines offer several benefits, such as structural stability, high safety, simple preservation, suitability for large-scale manufacturing, and improved immune specificity [12]. DEXs can contribute to tumor immunotherapy by modulating energy metabolism, influencing the inflammatory context, interacting between cells, delivering stimulatory signals, and so on. They can be transported to lymph nodes following their release to initiate specific cellular immunological responses [124]. Since DEXs have class I MHC, class II MHC, CD86, heat shock proteins (HSP) 70, and HSP90 chaperones, they have the ability to stimulate the activation of T lymphocytes [125]. The costimulation of released IL-2 and exosomal CD80 transfers the expression of the exosomal peptide MHC I to CD8 + T cells, thereby promoting their expansion and enhancing their immunity against tumors in vivo [126]. DEXs modulate the immune system’s reaction by directly presenting MHC-antigen complexes to T lymphocytes or indirectly to nearby APCs. Furthermore, they can directly stimulate NK cell activation and expansion by producing surface proteins such as natural killer group 2 member D ligands (NKG2DL) and IL-15R [124, 127]. In the first clinical trial, exosomes derived from autologous DCs developed as a melanoma vaccine. These DEXs were loaded with MAG-3 peptides and administered to late-stage melanoma patients. For 21 months, the exosomes demonstrated tolerability, accompanied by a minor inflammatory response at the injection site. Only one in fifteen patients had a specific T-cell response to melanoma antigens. Consequently, it exhibits low clinical efficacy in suppressing tumor growth [128]. However, the outcomes of the preclinical studies were satisfactory. For example, DEXs carrying melanoma antigen in the presence of Toll-like receptor three agonist poly (I: C) effectively triggered a strong activation of CD8 + T cells in a mouse model of melanoma. In addition, significant suppression of tumors and improved mouse survival were observed [129]. In another one, DEXs-loaded with a-galactosyl ceramide (aGC) and ovalbumin (OVA) were investigated. DEXs loaded with aGC have the ability to activate invariant NKT (iNKT) cells both in vivo and in vitro. Excitingly, only aGC on exosomes did not cause iNKT-cell anergy following a second injection, unlike soluble aGC. Subsequent to the natural immune response, they noted an increase of OVA-specific, IFNϒ-producing CD8 + T cells following inoculation with exosomal aGC/OVA, a result that surpassed that of exosomes devoting aGC. This led to reduced tumor growth and enhanced median survival of mice expressing [130].

#### Tumor-derived Exosmoses (TEXs)

Utilization of TEXs is also attractive because of their capacity to infiltrate and integrate into cells [131]. TEXs have proteins like MHCI, MHCII, CD81, CD54, and CD63, which facilitate exosome attachment and absorption by matching proteins on APCs. They also possess HSPs on their surface. HSPs stimulate APCs, establishing a natural connection between the immune system’s innate and acquired reactions. Further, HSPs possess potent adjuvant properties that augment the immunogenicity of TEXs and enhance the efficacy of cancer vaccines. Activated APCs presented TEXs, which are rich in tumoral antigens, to stimulate and activate T and B lymphocytes [132, 133]. Generally, TEXs exert a dual impact on the immune system, with the specific effect being amplified depending on the tumor stage [134]. In the early stages of cancer, EVs display tumor antigens that can stimulate and activate immune responses. But, during the advanced cancer stages, tumor cells and their EVs acquire immune-evasive traits. Nevertheless, the heterogeneity in the data may be attributed to the distinct experimental circumstances employed in each experiment and to the growing stage of the selected cell [135]. In melanoma, tumor development and migration were inhibited by TEXs containing tumor antigens. These TEXs were isolated from autologous tumor cells to regulate the T helper 1 (Th1) cell response [102, 136]. Park et al. demonstrated that melanoma cell-derived EVs coupled with synthetic bacterial vesicles (SyBV) resulted in a substantial reduction of tumor volume by 65%, although neither EVs nor SyBV alone exhibited any inhibitory effect. The substantial decrease in tumor proliferation resulted in improved survival, with mice inoculated with EV plus SyBV exhibiting a median survival duration of 24 days, as opposed to 18 days for the other groups [137]. Nevertheless, the immune response triggered by TEXs is relatively weak, resulting in insufficient antitumor effects. Hence, efforts are underway to develop vaccination systems, including artificially altered TEXs and TEX-loaded DCs with more immunogenicity [102].

In addition, exosomal vaccines stimulate the immune system and generate antigen-specific antibodies as a result of containing tumor antigens. Experiments done in vitro and in vivo have indicated that this method has the potential to harness the therapeutic capacity of exosomal vaccines and improve the immunotherapeutic impact in mice harboring B16-OVA [138]. The principal characteristics of TEX include their selective accumulation of tumor antigens, their systemic distribution, their membrane structure that enhances TEX binding and absorption, and the effective delivery of functionally competent TEX contents. In addition, TEXs can activate an extensive variety of T cell clones that react to several antigenic epitopes. Also, they could be readily isolated and purified using patients’ serum and tumor effusions [132]. Nevertheless, the exclusive use of TEXs did not yield particularly favorable outcomes due to their immunosuppressive properties. Consequently, many ways have been employed to enhance the efficacy of TEXs vaccines. TEXs can be manipulated, either genetically or non-genetically, to augment tumoral antigens, miRs, and immunostimulatory molecules, therefore directly promoting tumor cell apoptosis or facilitating their destruction by immune cells. An alternative method is to improve TEX vaccination by loading DCs with it. TEX-loaded DCs help turn naive CD8 + T cells into antigen-specific CTLs, activate NF-κB in macrophages, and help kill tumors by releasing tumor necrosis factor (TNF) [132, 133].

#### T cell-derived exosomes

CD4 + T cells, which are crucial immunological cells in the immune system, can stimulate CD8 + T cells to undergo differentiation into cytotoxic T lymphocytes (CTLs) through many pathways like secreting IL-2. IL-2 not only affects CD8 + T cells, but it also regulates CD4 + T cells’ effector functions and fate [139]. Simultaneously, CD4 + T cells also play a role in preserving and enhancing the antitumor activity of CTLs. The function of CD4 + T cells suggests that their exosomes can mediate the regulation of CD4 + T cell-mediated CD8 + T cell responses [139]. In addition, CD4 + T cells stimulate B cells to produce antibodies by releasing IL-2 or by interacting with cells to cell directly. So exosomes derived from CD4 + T cells are considered potential agents for mediating antitumor responses in cancer immunotherapy, given the current interest in this field [98]. These exosomes stimulate humoral immunity by boosting cell expansion, activation, and antibody generation. The uptake of exosomes, assisted by CD40L expression on their surface, causes the result [140]. Further, according to Shin et al., some miRs in exosomes made by CD4 + T cells can cause CTL-mediated antitumor responses in melanoma. Stimulation with IL-2 enhances these responses. They found that miR-10a-5p, miR-25-3p, miR-148a-3p, miR-155-5p, miR-215-5p, and miR-375 augmented granzyme B and IFN-ϒ mRNA levels in human CD8 + T lymphocytes compared to the control group. Moreover, miR-25-3p, miR-155-5p, miR-215-5p, and miR-375 substantially increased the protein production of IFN-ϒ, as assessed by flow cytometry in primary human CD8 + T cells [139]. CD8 + T cells are essential for specific tumor cell killing, acting as a vital component of anti-tumor immunity. Numerous studies indicate that exosomes produced from CD8 + T cells have clear anticancer properties in cancer therapy. For example, these exosomes contain a variety of cytotoxic compounds that either activate bystander T cells or stop mesenchymal cells from forming in the wound, showing how functionally important they are. Furthermore, activated CTL-derived exosomes have the ability to stimulate CTLs that have a low affinity for antigens, which helps support an extensive immune response [98, 141]. In an in vivo investigation utilizing a melanoma mouse model, the intratumoral delivery of exosomes produced from activated CD8 + T cells disrupted fibroblastic stroma-mediated tumor progression and metastasis [12]. In a study conducted by Jung et al., they generated IL-2-tethered exosomes from modified Jurkat T cells. Engineering These cells produce IL-2 on their plasma membrane utilizing a flexible linker, leading to an autocrine response. Following surface engineering of IL-2, the resulting exosomes’ miR profiles underwent substantial changes that activated CD8 + T cells and decreased the expression of PD-L1 in melanoma cells via differentially expressed miRs [142].

#### NK cell-derived exosomes

NK cells are a type of innate lymphoid cells that have a crucial role in fighting against cancer. Melanoma cells often evade immunotherapy by reducing the expression of MHC I and downregulating NKp30, NKp44, and NKG2D expression by NK cells. This phenomenon restricts the natural ability of NK cells to destroy cancer cells [143, 144]. However, recent investigations have demonstrated the significant anticancer effectiveness of exosomes generated from NK cells. The exosomes contain characteristic components of NK cells, such as perforin, granzymes, Fas ligand (FasL), and granulysin. NK cell-derived exosomes successfully eradicate cancer cells, like melanoma cells, by employing the proven direct killing mechanism [145–147]. The research by Zhu et al. showed that NK cell-derived exosomes can trigger apoptosis in B16F10 melanoma cells and hinder the development of melanoma xenografts. This might be associated with the presence of FasL and TNF-α in these exosomes [147]. Earlier studies have shown that NK cells are capable of secreting exosomes in both inactive and active states. Also, the exosomes generated from inactive NK cells contain FasL and perforin [148, 149]. Studies not only showed that NK cell-derived exosomes suppressed the growth and triggered the death of melanoma cells in vitro but also Suppress tumor proliferation in melanoma xenograft mice in vivo [147]. The complex dynamics of the TME impact the outcomes of immune cell therapy for malignant disorders and can diminish the efficacy of NK cell-based immunotherapy. Research has extensively shown that the acidic microenvironment around tumors may inhibit the secretion of perforin/granzymes from NK cells and block the interaction of Fas/FasL [150–152]. Acidity enhances the assembly and transportation of exosomes by attracting them and facilitating membrane fusion at a low pH level [153]. Hence, NK cell-derived exosome immunotherapy may offer benefits compared to therapy based on whole NK cells.

#### Other sources of exosomal vaccine

Researchers are now looking at macrophage-derived exosomes as a possible therapeutic exosomal vaccine for melanoma, along with TEXs, DEXs, and NK cell-derived exosomes. Exosomes produced from M1 macrophages have become well-known as significant players in cancer immunotherapy due to their tumoricidal behavior. M1 cell-derived exosomes that have taken up antigens can then transport them to CD4 + and CD8 + T cells [154, 155]. Furthermore, these exosomes can act as adjuvants for anticancer medications, enhancing their effectiveness in the treatment of cancer. M1 cell-derived exosomes have a particular ability to specifically target lymph nodes, wherein they are exclusively taken up by local macrophages and DCs. This results in the triggering of Th1 inflammatory immune reactions in the local context [156]. Fangfang Lv et al. showed that M1 OVA exosome, an exosomal vaccine constructed from OVA-stimulated M1 macrophages, can polarize TAMs into M1 phenotype by downregulating the Wnt signaling pathway, thereby enhancing the immune response and inhibiting tumor growth and metastasis [138]. In another study, researchers made changes to M1 cell-derived exosomes by including NF-kB p50, siRNA, and miR-511-3p. These RNA interferences are used to stimulate M1 polarization. Additionally, an IL4 receptor binding peptide (IL4-RBP) modifies the exosome surface to selectively target IL-4-Rs on M2 macrophages. M2 macrophages took in these changed exosomes, which then turned off the target genes, lowered the levels of M2 markers, and raised the levels of M1 markers [157]. Wang et al. performed a study where they used a new method to create chimeric exosomes. The researchers obtained exosomes from hybrid cells formed by the fusion of macrophages and tumor cells, with M1 macrophages phagocyting the nucleus of tumor cells. These possess a distinctive attribute as they exhibit tropism towards both lymph nodes and malignancies. The altered exosomes demonstrated the capacity to enhance T cell activation and multiplication through both direct exosome activation and activation mediated by APCs. The combined impact of these two pathways enhanced the effectiveness of ICIs in treatment. This demonstrates the potential of such altered exosomes as a helpful instrument for improving immunotherapeutic treatments [158]. These results strongly suggest that exosomes produced from modified M1 macrophages have significant potential for regulating the immune system and treating cancers.

### Removal of exosomes for melanoma therapy

Another approach for exosome-based cancer therapy involves removing exosomes from circulation or inhibiting their fusion and uptake by target cells [159]. so far, research in this area remains limited, emphasizing the need for further investigation. For instance, Zhou et al. found an increase in miR-494 in exosomes derived from the serum of melanoma patients. This finding was related to an increase in tumor progression. They demonstrated that blocking of exosome release by Rab27a leads to intracellular accumulation of miR-494, effectively preventing melanoma growth and metastasis. This suggests that inhibiting the transfer of miR-494 through exosomes could provide a novel strategy for developing miR-exosome treatments for melanoma [160]. In another study, researchers engineered Golgi apparatus-PDL1 exosome hybrid membrane-coated nanoparticles to block the production of PD-L1-bearing TEXs by targeting the Golgi apparatus in melanoma cells, as patients resistant to anti-PDL1 medication have elevated levels of TEXs containing PD-L1 [161, 162]. Additionally, Wang et al. demonstrated that eliminating PD-L1 expressing TEXs could be a viable approach to enhancing the effectiveness of ICI therapy [163].

### The challenges and limitations of using exosomes in cancer treatment

Even with all of the benefits of exosomes, accurately understanding them in relation to their therapeutic and delivery applications for medications still presents multiple challenges [22]. Moreover, clinical trials have only explored a limited number of exosome-based drug delivery systems for cancer treatment thus far. The fact that they are still in the early stages of clinical trials indicates that they still face numerous obstacles to overcome [93]. The primary limitations of exosome utilization restrict their clinical applicability as follows: [1] The in vivo functionality and safety of exosomes stay contentious. Because of their biological nature, it is important to take their safety into account when using exosomes as a means of transport. For instance, TEXs comprise tumor-promoting elements that facilitate tumor development, invasion, and metastasis, hence posing hazards related to immunosuppression or the acceleration of tumor progression. Consequently, additional clinical research on exosome treatment is required to bring out the safety and toxicity attributes of human trials [164, 2]. The production of exosomes in large quantities and sustainably is the primary obstacle for exosome therapy purposes. One of the most crucial elements is the donor cells’ selection and expansion. Despite advancements in cell culture technology, which now allow for the cultivation of up to 20,000 L of cells in steel and stainless bioreactors, the clinical application of exosomes remains challenging. The main causes are the high cost of scaling up, the precise and sensitive nature of cell culture, and the need to preserve donor cells’ genetic integrity [102]. During the mass production of exosomes, several challenges and obstacles arise, including: the quantity of exosomes extracted from biological fluids may be limited and variable [165]. Maintaining uniform quality in exosome preparations is essential for safety and efficacy, and establishing reliable quality control measures poses significant challenges [166]. Increasing production from laboratory to clinical scales frequently necessitates advanced bioreactor systems capable of sustaining the requisite conditions for exosome generation [167]. Extensive production techniques can be costly, increasing therapy expenses and restricting patient accessibility [168]. Exosomes may exhibit diminished stability during storage, potentially impacting their therapeutic efficacy [165, 3]. The change in membrane protein direction during membrane-disrupting procedures, such as medication delivery to exosomes, poses a risk of immune system identification and associated adverse effects. Although recently, organism generation-based in situ, exosome-based medication delivery methods have given researchers a new viewpoint. This strategy entails converting in vitro isolation, medication loading, and altered delivery into in situ release at the lesion’s location, thereby preventing the risk of changing exosome characteristics [169, 4]. The extraction and purification of the exosome is the other major problem. The use of conventional separation methods, such as extraction kits, tangential flow fractionation, and ultracentrifugation, is limited by the fact that they produce less-than-pure exosomes. At present, there are no established protocols for the separation of exosomes on a broad scale. Hence, there is a pressing need to devise a cutting-edge methodology that is highly efficient, superior quality, and cost-effective for the isolation of exosomes for drug delivery purposes [93, 5]. There is a lack of understanding of the processes governing the selective cellular absorption of exosomes and their intracellular dispersion patterns, leading to potential off-target effects [169, 6]. Among other challenges, we can mention the requirement for hybrid exosome designs for use in subsequent clinical uses, the high expense of post-drug acceptance, and a shortage of effective processes for defining the safety features of exosomal developed exosome-based platforms and methods for efficient drug delivery and disease treatment that are presently undergoing clinical study approvals [22]. Moreover, exosome therapy entails several risks linked to off-target consequences, such as unwanted biological reactions, alterations to the TME, elicitation of immunological responses, delivery of cytotoxic drugs to normal cells, and modulation of gene expression in recipient cells. These impacts may result in unexpected genetic alterations that might promote cancer or interfere with normal cellular functions. We don’t fully understand the long-term implications of exosome therapy, and while certain strategies aim to enhance targeting specificity, they might not completely eradicate off-target interactions. When employing exosome therapy, it is essential to evaluate these potential dangers to guarantee the treatment’s safety and effectiveness [168, 170–172].

**Table 1:** This table summarizes studies on exosome-based immunotherapy in melanoma

Type of Exosome-based immunotherapy	Intervention	Population description	Brief result	References
Exosomes as delivering vehicle	PEG-EV-Dox and simvastatin encapsulated into IL-13-functionalized long-circulating liposomes	B16F10 melanoma mice model	-Explicitly targeting the TAMs and cancerous cells -The significant suppression of various pro-angiogenic factors (VEGF, bFGF, and CD31) and an increase of the pro-apoptotic protein Bax	[173]
ASL-modified exosomes (AExs) as targeted delivering system	Dox-packed AExs	B16F10 melanoma mice model	-AExs specifically target melanoma locations through the cRGD peptide and integrin interaction -The proliferation of melanoma was significantly reduced, both in vitro and in vivo	[174]
cRGD engineered exosomes as targeted delivering system	cRGD-Exo/TPL	melanoma nude mouse model and A375 melanoma cell line	-Effectively suppressing the growth, invasion, and stimulation of apoptosis in laboratory conditions by disrupting the caspase pathway and mitochondrial routes, as well as modifying the distribution of the cellular cycle. -cRGD-Exo/TPL exhibited a remarkable capacity to target melanoma cancer in vivo and extended the half-life of TPL	[175]
Exosomes as delivering vehicle	AO	advanced melanoma cell lines Me 30,966	-A significant enhancement in the tumoricidal impact with low toxicity compared to free AO.	[176]
anti-CD20 aptamers modified exosomes as targeted delivering system	Adriamycin	WM266-4 and A375 melanoma cell lines and melanoma-bearing SCID mice (Targeting CD20 + stem cells in melanoma)	-The greatly augmented adriamycins effectiveness while minimizing systemic toxicity	[177]
Exosomes as carriers of anti-tumor antibodies	cGAMP and anti-CD40 and anti-PD-L1 molecules	B16F10-luc mice melanoma cell line	-Immunostimulation through the cGAMP, anti-CD40, and anti-PD-L1.	[178]
Exosomes as carriers of genetic information	BRAF siRNA	B16-F10 mice melanoma cell line	-siBRAF-mDexos has shown notable improvements in blood stability, biological compatibility, efficiency of absorption in B16-F10 cells, and cytotoxicity towards these cells in comparison with siBRAF -siBRAF-mDexos had a considerably more substantial suppressive impact on BRAF expression and increased the development of T-lymphocytes developement in melanoma cells.	[179]
Exosomes as carriers of genetic information	circular RNA RPS5	A375 and A2058 melanoma cell line	-Dose depended on reduction in cell survival and invasion. -Circular RPS5 achieves its tumor inhibitory effect by blocking miR-151a, which contributes to tumor development.	[180]
Exosomes as a tumor vaccine	Dex loaded with patient-specific neoantigens	B16F10 melanoma and MC-38 mouse models	-Dexs effectively inhibited tumor growth, prolonged life span, and prevented the spread of cancer to the lungs	[181]
Exosomes as a tumor vaccine	DEXs that included both antibodies against CTLA-4 and cancerous antigens	C57BL/6 mice with B16-F10 melanoma	-Dexs effectively trigger CTLs through the inhibition of CLTA-4 and simultaneously deliver tumor antigens	[182]
Exosomes as a tumor vaccine	genetically modified DEXs that combine antigen self-presentation and immunosuppression reversal (ASPIRE)	B16F10 melanoma mice model	-This nano vaccine significantly enhances the transport of antigens to lymphatic organs and directly stimulates both native and exhausted T lymphocytes	[99]
Exosomes as a tumor vaccine	DEXs, previously cultivated with OVA, which underwent modification with antibodies targeting CD3 and EGFR	C57BL/6J mice with B16-OVA melanoma	-These DEXs stimulated T cells, and they served as a connection between tumor cells and T cells by concurrently interacting with CD3 on T cell membranes and EGFR on tumor cells. -They successfully suppressed tumor relapse and spread by increasing the expression of PD-L1 in tumors.	[183]
Exosomes as a tumor vaccine	TEXs from B16-F1 melanoma cells containing CIITA and TRP2, a tumoral antigen.	C57BL/6 mice with melanoma	-CIITA-Exo caused an increase in the mRNA contents of inflammatory cytokines like TNF-α, the chemokine receptor CCR7, and the synthesis of IL-12, the stimulator cytokine of Th1 differentiation compared to the parental cell exosome. -CIITA-Exo exhibited a more robust anti-tumor response than the control exosome.	[184]
Exosomes as a tumor vaccine	genetically engineered TEXs from B16BL6 melanoma cells containing endogenous tumor antigens and immunostimulatory CpG DNA (CpG-SAV-Exo)	B16BL6 melanoma-bearing mice	-The administration of CpG-SV-Exo successfully stimulated the activation of DC2.4 cells and improved their ability to deliver tumor antigens.	[185]
Exosomes as tumor vaccine	Modified TEXs from B16 melanoma cells containing tumor- antigens and expressing M. tuberculosis antigen	B16 melanoma-bearing mice	-When altered exosomes injected to mice, they acquired resistance to both bacterial and tumor antigens. -The intratumoral administration of these exosomes effectively inhibited tumor growth compared to unmodified exosomes.	[186]
Exosomes as a tumor vaccine	HSP70-enriched TEXs from B16-F1 melanoma cells containing tumor- antigens	B16-MUC1 cell line and B16-MUC1 bearing C57BL/6 mice	-This exosome was found to enhance the levels of MHC-II and promote more significant amounts of IgG2a and IFN-ϒ.	[187]
Exosomes as a tumor vaccine	TEXs presenting OVA antigen and VSV-G	B16-OVA bearing C57BL/6 mice	-The simultaneous presence of OVA antigen and VSV-G on TEXs triggered a targeted immune response by enhancing the cross-presentation of antigen through a mechanism involving acidification of endosomes.	[188]
Exosome-like nanovesicles as a tumor vaccine	eNVs-FAP containing tumor-antigens	B16-F10 bearing mice	-The eNVs-FAP vaccination suppressed tumor growth by stimulating potent and targeted CTL responses towards both malignant cells and FAP + CAFs. -It altered the immunosuppressive features of TME in the melanoma cancer models.	[189]
Non-Immune cell-derived exosomes	Exosomes produced from cord blood stem cells	CHL-1 melanoma cell line	-These exosomes by displaying specific miRs, especially the miR-let-7 family, diminished the tumorigenicity and proliferation of cancer cells. -They induced a substantial reduction in survival rates for both cancer cells and lymphocytes derived from melanoma patients.	[190]
Non-Immune cell-derived exosomes	milk-derived exosomes	B16F10 melanoma cell lines	-miR-2478 found in milk exosomes can control melanogenesis in cancer cells by affecting Rap1a. This regulation occurs through the AKT-GSK3ß signaling pathway	[191]
Non-Immune cell-derived exosomes	Exosomes derived from modified MSCs to increase the production of TRAIL	B16F0 melanoma cell lines	-These exosomes caused cytotoxicity in melanoma cells through the expression of TRAIL.	[192]

*PEG; Polyethylene glycol*,* EV; Extracellular vesicle*,* Dox; Doxorubicin*,* IL; Interleukin*,* TAMs; Tumor associated macrophages*,* VEGF; Vascular endothelial growth factor*,* bFGF; Basic fibroblast growth factor*,* Bax; BCL2 associated X*,* ASL; Membrane anchor(BODIPY)-spacer(PEG)-targeted ligands(cyclic RGD peptides)*,* RGD; Cyclic arginylglycylaspartic acid*,* Exo; Exosome*,* TPL; Triptolide*,* AO; Acridine orange*,* SCID; Severe combined immunodeficiency*,* cGAMP; Cyclic guanosine monophosphate–adenosine monophosphate*,* PD-L1;Programmed death-ligand 1*,* si; Small interfering*,* RNA; ribonucleic acid*,* mDexos; Mature dendritic cells exosomes*,* miR; Micro ribonucleic acid*,* CTLA-4;Cytotoxic T-lymphocyte associated protein 4*,* CTLs; Cytotoxic T lymphocytes*,* EGFR; Epidermal growth factor receptor*,* OVA; Ovalbumin*,* TEXs; Tumor-derived exosomes*,* CIITA; Class II transactivator*,* TRP2;Tyrosinase-related Protein 2*,* mRNA; Messenger RNA*,* TNF-α;Tumor necrosis factor-α*,* CCR7;C-C chemokine receptor type 7*,* Th1;T helper1*,* SAV; Streptavidin*,* HSP70;70 kilodalton heat shock proteins*,* MHC-II; Major histocompatibility complex*,* IgG2a; Immunoglobulin G 2a*,* IFN*-ϒ;*Interferon gamma*,* VSV-G; G protein of vesicular stomatitis virus*,* eNVs; Exosome-like nanovesicles*,* FAP; Fibroblast activation protein-α*,* CAFs; cancer-associated fibroblasts*,* TME; Tumor microenvironment*,* Rap1a; Ras-related protein 1a*,* MSCs; Mesenchymal stem cells*,* TRAIL; TNF-related apoptosis-inducing ligand*

## Conclusion

Given that melanoma cancer is among the most lethal forms of cancer globally and that conventional treatments for these patients are often ineffective due to resistance, it is imperative to develop novel approaches for melanoma treatment. The utilization of exosomes for cancer treatment is a novel, promising approach owing to their inherent benefits. In this review, we have categorized the research conducted on the use of exosomes for melanoma treatment, which includes delivering chemotherapy drugs, cancer antigens as vaccines, immune system stimulants, and exosome expulsion. Exosomes are rapidly emerging as advanced nanomedicine tools in cancer immunotherapy. These therapeutic tools have gained significant attention in the past few decades due to their efficacy in delivering a wide range of chemical and natural medications. Modified exosomes that carry specific medications designed to target particular cancer cells provide greater advantages than conventional cancer treatments. However, this novel methodology also faces challenges previously mentioned, and it is still in its initial stages of development. In order to address these challenges, it is necessary to enhance cell culture technologies and construct bioreactors specifically designed to increase the production of exosomes [102]. The development of an innovative method for separating and purifying exosomes that is affordable, high-quality, and efficient is urgently needed [102]. On top of that, it is necessary to conduct further investigation into the optimal storage conditions for isolated exosomes derived from various cell sources. Overall, while there are still some obstacles to using exosomes in cancer immunotherapy, they provide an extremely efficient structure for this purpose. Increased efforts and further investigations will facilitate the advancement of this area in the future, ultimately revealing a novel path that will keep moving us forward in the realm of personalized cancer treatment. By utilizing exosomes’ unique properties, exosome therapy in personalized cancer medicine could change treatment approaches and improve patient outcomes and quality of life. For instance, by personalizing exosome-based therapies, clinicians can adjust the cargo according to the molecular profile of a patient’s tumor, potentially enhancing efficacy while reducing systemic adverse effects. Conducting clinical trials is essential for assessing the safety and efficacy of exosome therapies in cancer treatment. More data will help establish personalized application protocols and refine therapeutic strategies based on patient responses.

## Data Availability

No datasets were generated or analysed during the current study.

## Abbreviations

ACT: Adoptive cell therapy
aGC: a-Galactosylceramide
APCs: Antigen-presenting cells
BRAF: V-raf murine sarcoma viral oncogene homolog B1
CTLA-4: Cytotoxic T lymphocyte-associated protein 4
CTLs: Cytotoxic T lymphocytes
DC: Dendritic cell
DEX: DC-derived exosomes
DMFS: Distant metastasis-free survival
DNA: Deoxyribonucleic acid
DOX: Doxorubicin
EMA: European medicines agency
EVs: Extracellular vesicles
Fas L: Fas ligand
FDA: Food and drug administration
GM-CSF: Granulocyte-macrophage colony-stimulating factor
Gp100: Glycoprotein 100
HDI: High-dose IFN-α2b
HSP: Heat shock proteins
HSV-1: Herpes simplex virus 1
ICI: Immune checkpoint inhibitors
IFN: Interferon
IL: Interleukin
IL4-RBP: IL4 receptor binding peptide
iNKT: Invariant NKT
irAEs: Immune related adverse events
LAG-3: Lymphocyte activation gene 3
MAGE: Melanoma-associated antigen 1
MAPK: Mitogen-activated protein kinases
MART-1: Melanoma antigen recognized by T cells 1
MDSCs: Myeloid-derived suppressor cells
MEK: Mitogen-activated protein kinase kinase
MHC: Major histocompatibility complex
miRs: MicroRNAs
MM: Metastatic melanoma
mRNA: Messenger ribonucleic acid
MVBs: Multi vesicular bodies
NK: Natural killer
NKG2DL: Natural killer group 2 member D ligands
NRAS: Neuroblastoma ras viral oncogene homolog
ORR: Overall response rate
OS: Overall survival
OVA: Ovalbumin
PD-1: Programmed cell death protein 1
PDL-1: Programmed cell death-ligand 1
PEG: Polyethylene glycol
PFS: Progression-free survival
RFS: Relapse-free survival
RRM2: Ribonucleotide reductase regulatory subunit M2
siRNA: Small interfering RNA
SyBV: Synthetic bacterial vesicles
TAMs: Tumor-associated macrophages
TEXs: Tumor-derived exosmoses
Th1: T helper 1
TILs: Tumor-infiltrating lymphocytes
TME: Tumor microenvironment
TNF-α: Tumor necrosis factor-α
TPL: Triptolide
TRAIL: TNF-related apoptosis-inducing ligand
Tregs: Regulatory T cells
T-VEC: Talimogene laherparepvec
Tyr: Tyrosinase
VEGF: Vascular endothelial growth factor
